# Hydrogen sensors based on electrophoretically deposited Pd nanoparticles onto InP

**DOI:** 10.1186/1556-276X-6-392

**Published:** 2011-05-20

**Authors:** Jan Grym, Olga Procházková, Roman Yatskiv, Kateřina Piksová

**Affiliations:** 1 Institute of Photonics and Electronics, Academy of Sciences CR, v.v.i., Prague 8, Czech Republic; 2 Faculty of Nuclear Science and Physical Engineering, Czech Technical University in Prague, Prague, Czech Republic

## Abstract

Electrophoretic deposition of palladium nanoparticles prepared by the reverse micelle technique onto InP substrates is addressed. We demonstrate that the substrate pre-deposition treatment and the deposition conditions can extensively influence the morphology of the deposited palladium nanoparticle films. Schottky diodes based on these films show notably high values of the barrier height and of the rectification ratio giving evidence of a small degree of the Fermi level pinning. Moreover, electrical characteristics of these diodes are exceptionally sensitive to the exposure to gas mixtures with small hydrogen content.

## Introduction

Metal nanoparticles (MNPs) form a bridge between bulk materials and atomic or molecular structures. Bulk metals show constant size-independent physical properties, while the properties of MNPs are driven by their size, shape, and inter-particle distance. Surface properties are crucial because the number of surface atoms becomes significant as the MNP reaches the nanoscale limit [1]. III-V semiconductors have established their position in electronic devices thanks to their unique properties. As compared to silicon, they offer higher operating speeds, lower power consumption, or higher light emission efficiency. However, to fully exploit their properties, there is one key point remaining to be solved. III-V semiconductor structures suffer from a high density of surface/interface states causing so called Fermi level pinning (FLP) [2]. The mechanism responsible for the FLP at the metal-semiconductor interface has been a subject of a long-term discussion. We consider the disorder-induced gap state model stating that large energy deposition processes cause large disorder at the interface and thus a strong FLP [3]. The FLP leads to low Schottky barrier heights (SBH) on n-type III-Vs, which are metal independent when prepared by standard evaporation techniques [4]. Substantial improvements were reached by (i) incorporation of a thin native oxide [5], (ii) low-energy electrochemical deposition [6,7], and (iii) electroless plating [8].

In this article, we report on the preparation of Schottky barriers on InP substrates with increased SBHs by the electrophoretic deposition of palladium nanoparticles (NPs). We also demonstrate their application in hydrogen sensors. Regarding the group VIII transition metals, palladium and platinum are the two most preferred catalytic metals that have an outstanding capability of absorbing hydrogen [9]. Hydrogen molecules are adsorbed at the metal surface and partly dissociated into atoms. These atoms can diffuse through the metal to the interface with a semiconductor changing the SBH and accordingly the electrical properties of the structure. The hydrogen detection sensitivity and the Schottky barrier quality can be improved by reducing the metal grain size [10-12].

## Experimental

Pd NPs dispersed in isooctane solution were prepared by the reverse micelle technique [13]. Two reverse micelle solutions with identical molar ratio of water to AOT (sodium di-2-ethylhexylsulfosuccinate) were prepared. The first one was an aqueous solution of Pd(NH_3_)_4_Cl_2_, the second was an aqueous solution of hydrazine. Equal volumes of these solutions were mixed leading to the reduction of Pd(NH_3_)_4_Cl_2 _by hydrazine within the reverse micelles. As a result, Pd NPs with the diameters of 7 to 10 nm embedded in reverse micelles of AOT dispersed in isooctane were obtained.

The electrophoretic deposition from the colloid solution took place in a cell with two parallel electrodes. The upper electrode was made from high-purity graphite, the lower electrode was formed by an epi-ready InP substrate of n-type conductivity with the background concentration of about 6 × 10^15 ^cm^-3^. The substrates were cleaved from epi-ready wafers and all handling and depositions were conducted in a clean room facility. A back side ohmic contact to the InP substrate was formed by either rubbing liquid gallium with a tin rod or by vacuum evaporation of AuGeNi alloy. The distance between the electrodes was maintained at 1.5 mm. Pulsed DC voltage with a duty cycle of 50% and frequency of 10 kHz was applied for a selected period of time to deposit a Pd nanolayer. The pulsed voltage regime favors the deposition of individual nanoparticles over the deposition of nanoparticle clusters [14,15]. The deposition process was described in detail in [16]. Some of the substrates with deposited nanolayers were further annealed at 400°C in a vacuum of 10^-5 ^torr.

Layers of NPs were observed in JEOL JSM 7500F scanning electron microscope and by atomic force microscopy (AFM). Selected layers were contacted by the spots of a graphite colloid paint. These structures were further characterized by the measurement of current-voltage characteristics and their detection toward hydrogen was tested in a cell with a through-flow gas system.

## Results and discussion

We discuss the influence of (i) the final substrate surface treatment, (ii) the properties of the deposited colloid solution, (iii) the elecrophoretic deposition conditions (time, electrode polarity, applied voltage), and (iv) the post-deposition treatment of the layers (annealing at elevated temperatures) on the morphology of the deposited layers, their electrical properties, and their sensitivity toward hydrogen.

### Surface morphology

**Figure 1:**
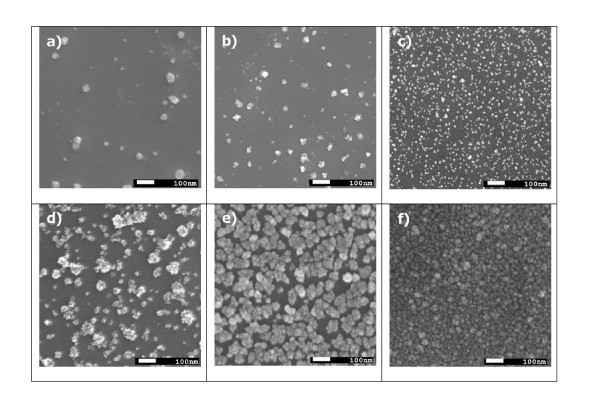
**SEM micrographs of Pd NPs deposited at different voltages and deposition times: (a) InP-Pd-06, 30 V, 1 h; (b) InP-Pd-05, 60 V, 1 h; (c) InP-Pd-04, 100 V, 1 h; (d) InP-Pd-07, 60 V, 4 h; (e) InP-Pd-09 100 V, 18 h; and (f) InP-Pd-25, 100 V, 3 × 10 h**. Magnification 60.000. The white scale bar corresponds to 100 nm. All substrates were treated in methanol before the deposition process.

First, the influence of the applied voltage during the electrophoretic deposition on the morphology of the deposited nanolayers was investigated. When a positive potential is applied to the InP substrate, very few Pd NPs are deposited. On the contrary, when a negative potential is applied to the substrate, a full coverage of the surface may be reached (Figure 1f). It can be concluded that the reverse micelles with Pd NPs in the solution are positively charged. From now on, all the samples discussed in this article were prepared with a negative potential applied to the substrate. The influence of the magnitude of the applied voltage for the layers deposited for 1 h at 30 to 100 V is demonstrated in Figure 1a, b, c. The higher the voltage, the higher the surface coverage and the smaller the size of deposited clusters. This can be described as follows. Sarkar et al. [17] found a striking analogy between the atomic film nucleation and growth by molecular beam epitaxy and electrophoretic deposition of silica microparticles. Let us assume that the electric field-in analogy with the supersaturation in epitaxial growth-is a driving force for the deposition process of Pd NPs. In epitaxial growth, higher supersaturation leads to a higher number of critical nuclei with a smaller size. Analogously, higher applied voltages and accordingly higher electric fields result in the deposition of a high density of individual Pd NPs.

Second, different surface treatments of InP substrates were performed. Conventional procedures for cleaning the substrates of III-V semiconductors consist in refluxing the substrate in a sequence of organic solvents such as trichloroethylene, acetone, methanol, and isopropyl alcohol to remove the contamination from heavy hydrocarbons, particles and heavy-atom contaminants [18]. The surface of epi-ready InP was (i) multiply rinsed in isooctane, (ii) treated in boiling methanol for 3 min, or (iii) treated in boiling isopropyl alcohol for 3 min. Different surface treatments significantly influenced the morphology of Pd nanolayers. While on the substrates treated in isooctane and isopropyl alcohol large clusters of Pd NPs were deposited, individual Pd NPs were observed on the substrates treated in methanol, which is in accordance with the conclusions of das Neves and de Paoli [19] that a single rinse in methanol can substitute a multiple rinse in different organic solvents, while a single rinse in isopropyl alcohol is insufficient for the preparation of a clean substrate surface.

Third, deposition times were varied to reach a different surface coverage. As expected, higher deposition times resulted in higher surface coverage (Figure 1c, d, e, f). Even at relatively long deposition times, the surface was not covered completely (Figure 1e). A full coverage was achieved by a multiple deposition (Figure 1f). This indicates that the colloid solution gradually depletes of Pd NPs. Moreover, at high deposition times (without changing the colloid solution), not only the Pd NPs are deposited, but also increased amounts of the surfactant (AOT) are observed on the surface by SEM.

Finally, some of the layers were annealed for 1 h at 400°C. This temperature was a compromise to remove AOT and not to cause damage to the InP substrate, which starts to decompose above 360°C. Luwang et al. investigated thermal properties of SnO_2_/AOT NPs in argon and air. They assigned exotermic peaks at 340°C to the decomposition of AOT [20]. Park et al. studied CdS/AOT and CdS/ZnS/AOT NPs in nitrogen and air and observed weight reduction due to the AOT removal from 220 to 380°C. The Fourier transform infrared spectroscopy showed that bands related to AOT were smaller on the samples subjected to 2-h treatment at 570°C compared to untreated samples; however, did not disappear completely [21]. Concerning the observation in SEM, after annealing, it was easier to observe individual Pd NPs, their round shape was truly visible, and no charging effects were experienced implying that remnants of the surfactant were partially removed. Also, adhesion of the layers was greatly enhanced. Non-annealed layers are susceptible to surface damage; improper handling leads to their partial removal. Besides, AFM observation is intricate as the AFM tip pushes the NPs toward the borders of the scanned area.

### Electrical properties and hydrogen detection

Two sets of samples were contacted by the graphite colloid paint to measure current-voltage (*I*-*V*) characteristics of the InP/Pd NPs/graphite structures and to characterize its capability of detecting hydrogen. Graphite can be deposited at room temperature and causes minimum disturbance to the semiconductor surface; it was reported to form good Schottky contacts on different semiconductors [22,23].

**Figure 2:**
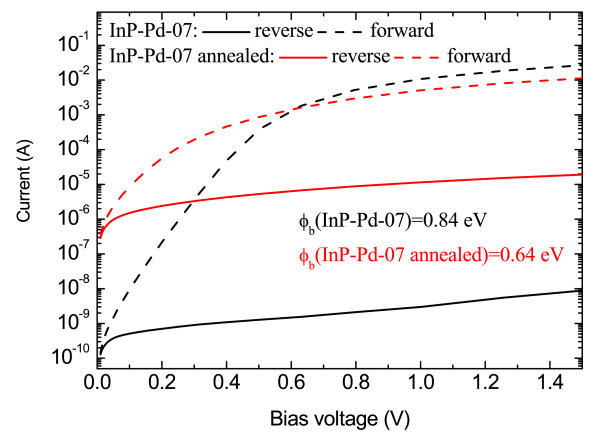
**Current-voltage characteristics of the sample InP-Pd-07 showing the influence of post-deposition annealing on the forward and reverse characteristics**.

**Figure 3:**
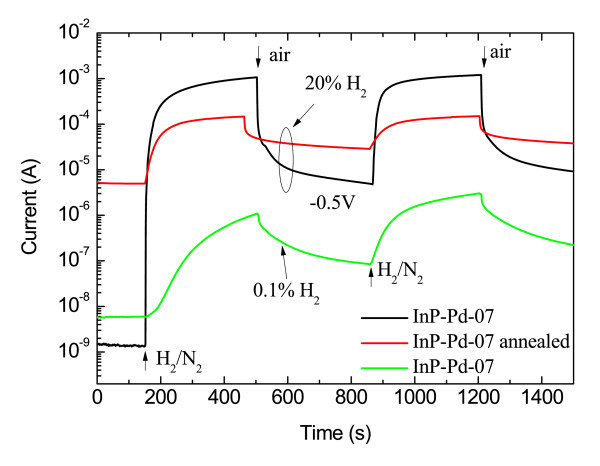
**Current transient characteristics for hydrogen detection showing the influence of annealing and the concentration of the testing gas mixture on the current of the diode which was reverse biased with the voltage of 0.5 V**.

The first set included structures from Figure 1a, b, c, d. The high values of SBH of 0.84-0.87 eV-in comparison with thermally evaporated Pd reaching 0.45 eV only-indicated a very low degree of Fermi level pinning. The value of SBH did not substantially vary with the deposition conditions. The influence of post-deposition annealing was more significant. Figure 2 shows *I*-*V *curves of the sample InP-Pd-07 from Figure 1d before and after annealing. Both the SBH and the rectification ratio *R *(defined as a ratio of the forward and reverse current at a given voltage) are considerably decreased after annealing. This decrease is tentatively assigned to the damage of the uncovered parts of the InP substrate and must be further investigated in detail. First experiments with hydrogen detection testing were performed with a mixture of H_2_/N_2 _containing 20% of H_2 _(Figure 3). A rapid current increase characterized by the sensing response *S *= 7.4 × 10^5 ^is observed for the sample InP-Pd-07. *S *= (*I*_H _- *I*_air_)/*I*_air_, where *I*_H _is a saturation current under the exposure to hydrogen and *I*_air _is the same for air. After annealing, the sensing response significantly drops to 0.29 × 10^2^. The same structure was later tested for 0.1% of H_2 _showing *S *= 1.8 × 10^2^.

**Figure 4:**
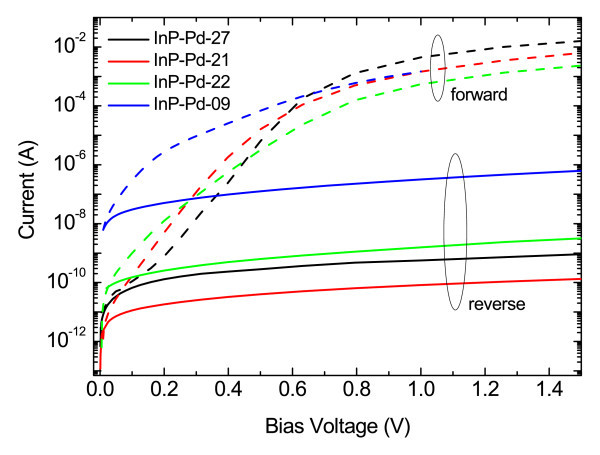
**Current-voltage characteristics of the diodes made on samples from Table 1**. The influence of the surface coverage on the forward and reverse characteristics is depicted.

The second set included samples that are summarized in Table 1. Their I-V curves are shown in Figure 4. All samples were prepared on methanol-treated substrates at 100 V and tested for low percentage H_2_/N_2 _mixture of 0.1%. The deposition time was varied to change the surface coverage. All the investigated parameters reach its optimum values when the surface is partly covered by individual Pd NPs (1-hour deposition in Figure 1c). An outstanding value of the sensing response of 4.8 × 10^5 ^was achieved. This value is at least by two orders of magnitude higher than for any other Schottky diode-based sensor on III-V semiconductors. When shorter deposition times below 30 min are applied, the sensing response quickly decreases. Longer deposition times and full surface coverage bring results similar to those published by other groups for 0.1% H_2_/N_2 _mixtures [11,12].

**Table 1:** Summary of the deposition conditions and electrical characteristics of the samples prepared on methanol-treated substrates

Sample	Time (h)	Voltage (V)	*R *at 1.5 V	*ϕ*_b _(eV)	*S *at 0.1%H_2_
InP-Pd-27	0.5	100	1.8E7	0.93	5.0E4
InP-Pd-21	1	100	4.8E7	0.95	4.8E5
InP-Pd-22	2	100	0.7E7	0.88	1.6E4
InP-Pd-09	18	100	1.0E4	0.74	4.9E2

*R *is the rectification ratio, *ϕ*_b _is the Schottky barrier height, and *S *is the sensing response

**Figure 5:**
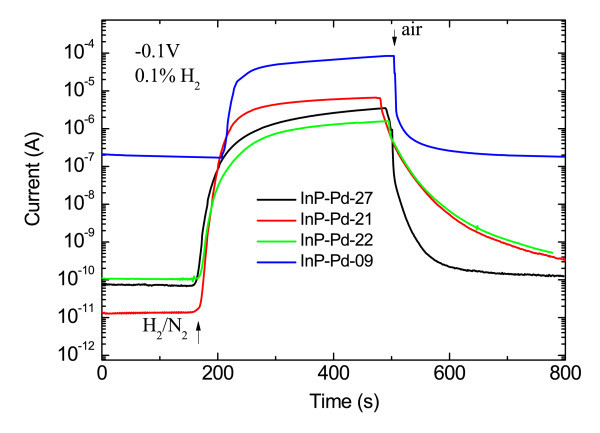
**Current transient characteristics of the diodes made on samples from Table 1, which were exposed to 0.1% H_2_/N_2 _mixture**. The influence of the surface coverage on the current of the diode which was reverse biased with the voltage of 0.1 V is shown.

The mechanism of the detection is not discussed in detail and can be shortly described as follows. The hydrogen molecules are absorbed and dissociated at Pd surface; atomic hydrogen rapidly diffuses to the Pd/InP interface, where the dipole layer develops. Subsequently, the Schottky barrier height decreases and the electric current increases [12] (Figure 5).

## Conclusions

Preparation of Pd NPs by the reverse micelle technique and their electrophoretic deposition onto InP substrates were discussed. We were able to vary the surface morphology of the films formed by Pd NPs from several individual NPs on the surface to its full coverage. Variety of morphologies was achieved by changing the substrate pre-deposition treatment and the deposition conditions. Schottky diodes based on these films showed notably high values of the barrier height up to 0.95 eV and of the rectification ratio up to 4.8 × 10^7 ^giving evidence of a small degree of the Fermi level pinning. Moreover, electrical characteristics of these diodes were exceptionally sensitive to the exposure to gas mixtures with small hydrogen content. An outstanding value of the sensing response of 4.8 × 10^5 ^was achieved for the 0.1% H_2_/N_2 _mixture pointing to the bright prospects of these structures in extremely sensitive hydrogen sensors.

## Abbreviations

AFM: atomic force microscopy; FLP: Fermi level pinning; NPs: nanoparticles; SBH: Schottky barrier height.

## Competing interests

The authors declare that they have no competing interests.

## Authors' contributions

JG drafted and wrote the manuscript, designed the electrophoretic deposition experiments and participated in the interpretation of the measured data and the project coordination. OP conceived the study and participated in the design of experiments. RY conducted electrical measurements and participated in the interpretation of the measured data, KP was responsible for the preparation of Pd NPs and SEM characterization. All authors read and approved the final manuscript.

## References

[B1] Hossam H Chemical sensors based on molecularly modified metallic nanoparticles J Phys D 2007 40 23 7173 7186 10.1088/0022-3727/40/23/S01

[B2] Hasegawa H Akazawa M Interface models and processing technologies for surface passivation and interface control in III-V semiconductor nanoelectronics Appl Surf Sci 2008 254 24 8005 8015 10.1016/j.apsusc.2008.03.051

[B3] Hasegawa H Ohno H Unified disorder induced gap state model for insulator-semiconductor and metal-semiconductor interfaces J Vac Sci Technol B 1986 4 4 1130 1138 10.1116/1.583556

[B4] Hokelek E Robinson GY A study of Schottky contacts on indium phosphide J Appl Phys 1983 54 9 5199 5205 10.1063/1.332745

[B5] Wada O Majerfeld A Robson PN InP Schottky contacts with increased barrier height Solid-State Electron 1982 25 5 381 387 10.1016/0038-1101(82)90123-X

[B6] Hasegawa H Inteface-controlled Schottky barriers on InP and related materials Solid-State Electron 1997 41 10 1441 1450 10.1016/S0038-1101(97)00087-7

[B7] Hasegawa H Fermi level pinning and Schottky barrier height control at metal-semiconductor interfaces of InP and related materials Jpn J Appl Phys 1999 38 2B 1098 1102

[B8] Chen HI Chou YI Chu CY A novel high-sensitive Pd/InP hydrogen sensor fabricated by electroless plating Sens Actuators B 2002 85 1-2 10 18 10.1016/S0925-4005(02)00044-8

[B9] Carturan G Cocco G Facchin G Navazio G Phenylacetylene hydrogenation with Pd, Pt and Pd-Pt Alloy catalysts dispersed on amorphous supports - effect of Pt/Pd ratio on catalytic activity and selectivity J Mol Catal 1984 26 3 375 384 10.1016/0304-5102(84)85111-1

[B10] Sato T Uno S Hashizume T Hasegawa H Large Schottky barrier heights on indium phosphide-based materials realized by in-situ electrochemical process Jpn J Appl Phys 1997 36 3B 1811 1817

[B11] Chou YI Chen CM Liu WC Chen HI A new Pd-InP Schottky hydrogen sensor fabricated by electrophoretic deposition with Pd nanoparticles IEEE Electron Device Lett 2005 26 2 62 65

[B12] Kimura T Hasegawa H Sato T Hashizume T Sensing mechanism of InP hydrogen sensors using Pt Schottky diodes formed by electrochemical process Jpn J Appl Phys 2006 45 4B 3414 3422 10.1143/JJAP.45.3414

[B13] Chen D-H Wang C-C Huang T-C Preparation of palladium ultrafine particles in reverse micelles J Colloid Interface Sci 1999 210 1 123 129 10.1006/jcis.1998.5795 9924114

[B14] Naim MN Iijima M Kamiya H Lenggoro IW Electrophoretic packing structure from aqueous nanoparticle suspension in pulse DC charging Colloids Surf A 2010 360 1-3 13 19 10.1016/j.colsurfa.2010.01.057

[B15] Naim MN Iijima M Sasaki K Kuwata M Kamiya H Lenggoro IW Electrical-driven disaggregation of the two-dimensional assembly of colloidal polymer particles under pulse DC charging Adv Powder Technol 2010 21 5 534 541 10.1016/j.apt.2010.02.004

[B16] Zdansky K Zavadil J Kacerovsky P Lorincik J Vanis J Kostka F Cernohorsky O Fojtik A Reboun J Cermak J Electrophoresis deposition of metal nanoparticles with reverse micelles onto InP Int J Mater Res 2009 100 9 1234 1238

[B17] Sarkar P De D Yamashita K Nicholson PS Umegaki T Mimicking nanometer atomic processes on a micrometer scale via electrophoretic deposition J Am Ceram Soc 2000 83 6 1399 1401 10.1111/j.1151-2916.2000.tb01400.x

[B18] Ingrey S III-V-Surface processing J Vac Sci Technol A 1992 10 4 829 836 10.1116/1.577680

[B19] Das Neves S De Paoli MA Monitoring the organic cleaning process of Inp crystals by contact-angle measurement Semiconductor Sci Technol 1994 9 9 1719 1721 10.1088/0268-1242/9/9/023

[B20] Luwang MN Ningthoujam RS Singh NS Tewari R Srivastava SK Vatsa RK Surface chemistry of surfactant AOT-stabilized SnO2 nanoparticles and effect of temperature J Colloid Interface Sci 2010 349 1 27 33 10.1016/j.jcis.2010.05.037 20557894

[B21] Park K Yu H Chung W Kim B-J Kim S Effect of heat-treatment on CdS and CdS/ZnS nanoparticles J Mater Sci 2009 44 16 4315 4320 10.1007/s10853-009-3641-2

[B22] Tongay S Schumann T Hebard AF Graphite based Schottky diodes formed on Si, GaAs, and 4H-SiC substrates Appl Phys Lett 2009 95 22 222103 222103 10.1063/1.3268788

[B23] Tongay S Schumann T Miao X Appleton BR Hebard AF Tuning Schottky diodes at the many-layer-graphene/semiconductor interface by doping Carbon 2011 49 6 2033 2038 10.1016/j.carbon.2011.01.029

